# Profile of eye-related emergency department visits in Ontario – a Canadian perspective

**DOI:** 10.1186/s12886-023-02999-x

**Published:** 2023-07-10

**Authors:** Keean Nanji, Husayn Gulamhusein, Yasmin Jindani, David Hamilton, Kourosh Sabri

**Affiliations:** 1 Department of Surgery, Division of Ophthalmology, McMaster University, 1200 Main Street West, 3V2, L8N 3Z5 Hamilton, ON Canada; 2 Department of Health Research Methods, Evidence and Impact, McMaster University, 1280 Main Street West, Hamilton, ON Canada; 3 McMaster Paediatric Eye Research Group (McPERG), McMaster University, Hamilton, ON Canada; 4 Division of Emergency Medicine, Department of Medicine, McMaster University, Hamilton, ON Canada

**Keywords:** Epidemiology, Ophthalmology, Trauma, Emergency medicine

## Abstract

**Background:**

Understanding the epidemiology of ophthalmic presentations to emergency departments can help guide resource allocation, medical education programs, and optimize the patient experience. The purpose of this investigation was to summarize and assess the urgency of ophthalmic presentations in emergency departments (EDs) in Ontario, Canada over a 5-year period.

**Methods:**

This was a multicentered retrospective review of all patient presentations to EDs in Ontario between January 1st, 2012, to December 31st, 2017. Presentations were included if patients had an ophthalmic related ICD-10 code as their primary problem prompting ED presentation.

**Results:**

A total of 774,057 patients patient presentations were included across the pediatric (149,679 patients) and adult (624,378 patients) cohorts. The mean (SD) age at presentation was 47.4 (17.9) years, and 6.54 (5.20) in the adult and pediatric cohorts respectively. Of the total presentations, 256,776 (33.1%) were due to a trauma related presentation. Problems pertaining to Cornea and External disease were the most common reason for presentation (51.0% of cases). Of all presentations, 34.1% were classified as either ‘emergent’ or ‘likely emergent’; the remaining presentations were either ‘non-emergent’ (39.5%) or the urgency ‘could not be determined’ (26.4%). The three most frequent presentations were due to conjunctivitis (121,175 cases or 15.7%), ocular foreign bodies (104,322 cases or 13.5%), and corneal / conjunctival abrasions (94,554 cases of 12.2%).

**Conclusions:**

This investigation summarizes all ophthalmic presentations to EDs in Ontario, Canada over a 5-year period. The results of this investigation can help guide ophthalmic related knowledge translation. Additionally, these results highlight that in Canadian EDs, a significant proportion of ophthalmic presentations are nonurgent; systems level efforts to improve access for eye-related complaints to healthcare professionals outside of the ED can help facilitate improved resource allocation. As we emerge from the COVID-19 pandemic, optimising the structure of patient care access is crucial to help alleviate the pressure from overburdened EDs while effectively meeting patient healthcare needs.

**Supplementary Information:**

The online version contains supplementary material available at 10.1186/s12886-023-02999-x.

## Introduction

### Background

Emergency medicine (EM) healthcare providers are tasked with the challenge of providing high-quality patient care while treating a broad range of pathologic conditions.[1, 2] Previous investigations have demonstrated that between 1.5% and 3.4% percent of total visits to the emergency department are due to eye-related conditions.[3, 4] Despite this, ophthalmology training in medical schools is very limited in Canada and around the world.[5] Presently, 50% of Canadian medical school clerkship programs report a mandatory ophthalmology rotation;[6] of those that do have a rotation, the median duration is 1-week.[6] In the United States, only 18% of medical schools report a mandatory ophthalmology clerkship rotation.[7] As a result, 64% of first-year residents reported “too little” or “no” ophthalmology exposure in medical school. [8] Previous investigations have demonstrated similar trends when evaluating the ophthalmic competencies of emergency medicine physicians; a Canadian investigation reported that only 39% of emergency medicine referrals to ophthalmology had a correct diagnosis.[9] Similarly, many emergency medicine physicians do not feel comfortable with their ophthalmic exam in the United Kingdom,[10] and the United States.[11].

It can be challenging to increase ophthalmology exposure in medical education given the exponential increase in medical knowledge and current crowding of medical curriculums. [12] As such, understanding the epidemiology of ophthalmic presentations to the emergency department is crucial to guide ophthalmic training in medical school and residency. Medical education in Canada and around the world is undergoing a major transformation to a competency-based system.[13] The competencies deemed necessary for learners are based on evidence and practice patterns. Presently, there exists a lack of evidence examining the epidemiology and ophthalmic related presentations to Canadian emergency department.

In an effort to address this gap, the purpose of this investigation is to summarize and perform a descriptive analysis of patients with primary ophthalmic complaints presenting to emergency departments (EDs) in Ontario, the largest province in Canada, over a 5-year period.

## Methods

### Study design and time period

Aggregate population-based data was obtained for the purposes of this study from the Canadian Institute for Health Information (CIHI). CIHI is a not-for-profit governmental organization tasked with collecting and collating health-related data across the country to facilitate research and advancement in the healthcare sector. A list of all International Classification of Diseases 10th Revision (ICD-10) codes associated with ophthalmic pathology was created (*Supplementary Table 1*), and aggregate data related to patients with an ophthalmic ICD-10 code listed as their primary presenting problem within the database was collected.

The Hamilton Integrated Research Ethics Board provided ethical approval for the study design and execution. While data was collected from pediatric patients, there was no direct involvement of minors in this investigation. The need for informed consent from all included participants and/or their legal guardian (s) was waived by the Hamilton Integrated Research Ethics Board. All research was carried out following the tenets of the Declaration of Helsinki and the Good Clinical Practice guidelines.

### Study setting, timeline, and population

This was a multicentered retrospective review. Data related to all patients presenting to an Ontario, Canada emergency department from January 1st, 2012, to December 31st, 2017, with an ICD-10 visit diagnostic code related to an ophthalmic problem was collected. Patients of all ages were included. There were no exclusion criteria.

### Outcome measures

The primary outcome measure was the frequency of primary presentation to the emergency department due to an ophthalmic complaint, stratified by complaint type and threat to vision. Secondary outcome measures included traumatic ophthalmic complaints, complaint type categorized by ophthalmic subspeciality, Canadian Triage and Acuity Scale (CTAS) level for each presentation, patient referral source, patient access to primary care and whether the injury occurred at the workplace.

### Data analysis

All ophthalmic diagnoses were classified into one of three levels of acuity utilizing the classification regarding threat to vision of each ophthalmic ICD code proposed by Channa et al.[14] Diagnoses were classified as either (a) *emergent or likely emergent*, (b) *unlikely to be emergent or nonemergent* or (c) *could not be determined*. Diagnoses were also classified into the most relevant subspecialty based on consensus amongst two senior ophthalmology residents (HG, KN). The subspecialty categories included: (a) cornea and external disease, (b) general ophthalmology, (c) oculoplastics and orbit, (d) retina and vitreous, (e) uveitis and ocular inflammation, (f) neuro-ophthalmology and strabismus, (g) glaucoma.

Descriptive statistics were generated using Statistical Product and Service Solutions (IBM, Armonk, NY, Version 27).

## Results

From 2012 to 2017, a total of 774,057 patients presented to an emergency department in Ontario with an ophthalmic complaint prompting ED presentation. Of these, 624,378 were patients classified as adults (ages 18 or older), and 149,679 classified as pediatric (ages 17 or younger). In the adult population, the mean (SD) age at presentation was 47.4 (17.9) years, and 57.0% of patients were male. In the pediatric cohort, the mean (SD) age was 6.54 (5.20) and 54.9% were male. Of the total presentations, 224,915 (36.0%) and 31,861 (21.3%) were due to a trauma related presentation in the adult and pediatric cohort respectively. The full summary of the characteristics of included participants is displayed in Table 1.

**Table 1:** Summary of Included Patients

Variable	Number of Patients	Relative Percentage
Total Included Population	774,057	N/A
Total Adult Population	624,378	80.7%
Total Pediatric Population	149,679	19.3%
Males	438,059	56.6%
Females	335,942	43.4%
Unidentified Genders	45	0.0%
Other Genders	10	0.0%
Mean Age in Adult Population (SD)	47.4 (17.9)	N/A
Mean Age in Pediatric Population	6.54 (5.2)	N/A
Mean Triage Level in Adult Population (SD)	3.3 (0.8)	N/A
Mean Triage Level in Pediatric Population	3.52 (0.7)	N/A
Number of Trauma Cases in Adult Population	224,915	36.0%
Number of Trauma Cases in Pediatric Population	31,861	21.3%

### Presenting complaints – adult cohort

In the adult cohort, the most frequent presenting complaints during the study period were ocular foreign bodies (100,152 cases or 16.0%), conjunctivitis (86,015 cases or 13.8%), corneal / conjunctival abrasions (83,480 cases of 13.4%), visual disturbances (50,392 or 8.1%), and hordeolum/styes (29,715 cases or 4.8%). Visual disturbances included, but were not limited to, complaints of flashing lights, floaters, and scintillating scotomas. Cases were classified as emergent for 230,684 presentations (36.9%), as non-emergent for 220,845 presentations (35.4%) and could not be determined in 172,849 presentations (27.7%). Table 2 displays the 10 most frequent presenting complaints along with the acuity classification of each diagnosis and Supplemental Table 1 displays the full list of all presenting complaints with the corresponding classification of acuity.

**Table 2:** The 10 Most Frequent Ophthalmic Problems in Adult Cohort

ICD-10 Code	Description	Likely Emergent?	Frequency of Primary Problem	Percentage of Primary Problem
T159	Foreign body on external eye, unspecified part	Yes	100,152	16.04%
H109	Conjunctivitis, unspecified	No	86,015	13.78%
S050	Injury of conjunctiva and corneal abrasion without mention of foreign body	Yes	83,480	13.37%
H539	Visual disturbance, unspecified	Could not determine	50,392	8.07%
H000	Hordeolum or Stye	No	29,715	4.76%
H113	Conjunctival haemorrhage	No	24,778	3.97%
H571	Ocular pain	Could not determine	23,759	3.81%
H578	Other specified disorders of eye and adnexa	Could not determine	23,286	3.73%
S059	Injury of eye and orbit, unspecified	Could not determine	11,608	1.86%
H332	Serous retinal detachment	Yes	10,789	1.73%

### Presenting complaints – pediatric cohort

In the pediatric cohort, the most frequent presenting complaints were conjunctivitis (35,160 cases or 29.16%), corneal/conjunctival abrasion (11,074 cases of 9.18%), acute inflammation of the orbit (abscess, cellulitis, osteomyelitis etc.) (5,843 cases or 4.85%), hordeolum (5,800 or 4.85%) and other specified disorders of the eye and adnexa (5,335 or 4.42%). Of the pediatric presentations, 33,629 (22.5%) were classified as emergent, 84,851 (56.7%) were classified as non-emergent and 31,199 (20.8%) were classified as could not be determined. Table 3 displays the 10 most frequent presenting complaints with their acuity classification and Supplemental Table 2 displays the full list of all pediatric presenting complaints with the corresponding classification of acuity.

**Table 3:** The 10 Most Frequent Ophthalmic Problems in the Pediatric Cohort

ICD-10 Code	Description	Likely Emergent?	Frequency of Primary Problem	Percentage of Primary Problem
H109	Conjunctivitis, unspecified	No	35,160	29.16%
S050	Injury of conjunctiva and corneal abrasion without mention of foreign body	Yes	11,074	9.18%
H050	Acute inflammation of orbit (abscess, cellulitis, osteomyelitis, periostitis, tenonitis)	Yes	5,843	4.85%
H000	Hordeolum and other deep inflammation of eyelid	No	5,800	4.81%
H578	Other specified disorders of eye and adnexa	Could not determine	5,335	4.42%
B309	Viral conjunctivitis (unspecified)	No	4,754	3.94%
S059	Injury of eye and orbit, unspecified	Could not determine	4,555	3.78%
T159	Foreign body on external eye, unspecified part	Yes	4,170	3.46%
H108	Other conjunctivitis	No	3,836	3.18%
H101	Acute atopic conjunctivitis	No	3,306	2.74%

### Trauma related presentations – adult cohort

The three most frequent traumatic presenting complaints were ocular foreign bodies (100,152 cases; 44.5% of all trauma presentations), corneal / conjunctival abrasions (83,480; 37% of all adult trauma presentations), and unspecific injuries of the eye and orbit (20,743; 9.2% of adult trauma presentations). Of note, a penetrating wound of eyeball with foreign body occurred in 1269 cases and ocular laceration and rupture with prolapse or loss occurred in 132 cases. Tables 4 and 5 summarize the 10 most frequent trauma related ocular presentations in the adult and pediatric population respectively. Supplemental Tables 3 and 4 display the full list of trauma related presentations in the adult and pediatric cohorts.

**Table 4:** Most Frequent Trauma Related Ophthalmic Presentations in Adult Population

ICD-10 Code	Code Description	Frequency of Ophthalmic Problem	Percentage of Ophthalmic Problem
T159	Foreign body on external eye, or Cornea	100,152	44.50%
S050	Corneal abrasion/Injury of conjunctiva	83,480	37.10%
S059	Injury of eye and orbit, unspecified (includes injury of eye NOS)	20,743	9.20%
T264	Ocular/Orbital burns	7,920	3.52%
S0110	Eyelid Abrasion and Laceration	5,487	2.40%
S051	Contusion/Blunt Trauma Injuries of the Eye and Orbit	4,479	2.00%
S0110	Open wound of eyelid, uncompl.	4,436	1.97%
S051	Contusion of eyeball and orbital tissues (includes corneal contusion, traumatic hyphaema)	3,449	1.53%
T264	Burn of eye and adnexa, part unspecified (includes welder’s flash)	3,198	1.42%
S055	Penetrating wound of eyeball with foreign body	1,269	0.56%

**Table 5:** Most Frequent Trauma Related Ophthalmic Presentations in the Pediatric Cohort

ICD-10 Code	Code Description	Frequency of Ophthalmic Problem	Percentage of Ophthalmic Problem
S050	Corneal abrasion/Injury of conjunctiva	11,074	34.76%
S059	Injury of eye and orbit, unspecified (includes injury of eye NOS)	4,555	14.30%
T159	Foreign body on external eye, part unspecified	4,170	13.09%
S0110	Open wound of eyelid, uncompl.	3,112	9.77%
S058	Other injuries of eye and orbit (includes lacrimal duct injury)	2,588	8.12%
T150	Foreign body in cornea	1,676	5.26%
S002	Other superficial injuries of eyelid and periocular area	1,284	4.03%
S051	Contusion of eyeball and orbital tissues (includes corneal contusion, traumatic hyphaema)	984	3.09%
S001	Contusion of eyelid and periocular area	463	1.45%
T151	Foreign body in conjunctival sac	430	1.35%

### Trauma related presentations – pediatric cohort

The three most frequent traumatic presenting complaints in the pediatric cohort were corneal abrasion/conjunctival abrasion (11,074; 34.8% of pediatric trauma cases), injury of the eye and orbit, unspecified (4,555 or 14.30% of pediatric trauma cases), foreign body on the external eye (4,170 or 13.1% of pediatric trauma cases). Table 4 displays the frequency of the 10 most common trauma related presentations with the full list in Supplemental Table 4.

### Presentations by subspecialty

Of patients presenting to the ED across the pediatric and adult cohorts, 51.0% of presentations (394,750 cases) were due to a primary complaint relating to Cornea and External disease. The remaining breakdown of presentations by subspecialty were due to General Ophthalmology (21.64%), Oculoplastics and Orbit (17.1%), Retina and Vitreous (5.5%), Uveitis and Ocular Inflammation (2.1%), Neuro-ophthalmology and Strabismus (2.1%) and Glaucoma (0.8%).

### Referral source

The referral sources for patients to the ED sorted in order of frequency were (1) self/family member, caretaker, guardian (90.9% of presentations), (2) Ambulatory care service (facility based) (3.5% of presentations), and (3) private practice (e.g. physician, midwife, chiropractor) (3.1% of presentations).

### Primary care access

Patients had access to a family physician in 85.9% of cases, did not have access to a primary care provider in 9.5% of cases, had access to a primary care provider that wasn’t their family physician (including a family health team or walk-in clinic) in 1.1% and whether the patient had access to a primary care physician was not reported in 3.4% of cases.

### Workplace injury

Of included presentations, 96.2% were not due to a workplace injury, 2.5% were due to a workplace injury and whether the injury occurred at a workplace was not reported in 1.3% cases.

## Discussion

This investigation provides a summary of all eye-related presentations to EDs in Ontario, Canada, over a 5-year period. Nearly 800,000 presentations have been summarized and to our knowledge, this is the largest study to examine Canadian ED ophthalmologic visits. Presentations relating to cornea and external diseases were the most frequent across all cases and across trauma related presentations and as such should be a substantial focus of ED resident training. Additionally, of all presentations, only 34.1% were classified as either ‘emergent’ or ‘likely emergent’; the remaining presentations were either ‘non-emergent’ (39.5%) or the urgency ‘could not be determined’ (26.4%). Recognizing that a substantial proportion of presentations were nonemergent and non-vision threatening can assist policymakers better direct care out of emergency departments and improve resource allocation.

Only approximately one third of presentations to the ED were ‘emergent’ or ‘likely emergent’. The remaining presentations were either non-urgent and likely did not require emergency level care or the urgency could not be determined solely from the ICD code and patients likely would have been suited seeking care from an optometrist rather than an emergency medicine physician. Previous investigations have demonstrated that optometrists more accurately diagnose ophthalmic conditions compared to emergency medicine physicians. [9, 15, 16] Conjunctivitis and styes/hordeolum were the second and fifth most common diagnoses in the adult population and the first and fourth most common diagnoses in the pediatric population. These two conditions alone were responsible for over 155,000 visits to the emergency department in Ontario over the 5-year study period. These conditions and the other non-urgent conditions pose minimal threats to emergent vision loss and could be managed in non-emergent settings such as by an optometrist, primary care provider, or in an urgent care.[14] This would not only have economic benefits due to the higher cost of treating these conditions in the ED, but in the case of seeing an optometrist, would also result in patients receiving care from a healthcare professional with greater training in managing eye-related conditions.[9, 15, 16] This finding is similar to previous literature which has demonstrated that between 44% and 60% of ophthalmic presentations to the ED were for nonemergent problems.[14, 17] In this investigation, 87% of patients reported having access to a primary care provider; systems level changes to help redirect patients with nonurgent presentations to more appropriate care can help with ED overcrowding.[18, 19] The results of this investigation are of particular importance given the increase in burnout seen by ED providers from the COVID-19 pandemic.[20, 21] As we enter into the post-COVID-19 era, restructuring acute care delivery systems may be an effective approach to help more effectively meet patients’ needs while reducing the burden on already overwhelmed EDs.

Over 50% of presentations to the ED were due to problems relating to Cornea and External disease. Ophthalmic problems relating to retina, neuro-ophthalmology and glaucoma consisted of a combined less than 10% of presenting complaints. These results are in-keeping with previous investigations which have similarly demonstrated anterior segment pathology to be most frequent impetus for patient presentation to the ED.[17, 22, 23] As such, approaches to frequent corneal and external eye conditions should be a significant focus of emergency medicine teaching. Recognizing emergent vision threatening presentations and when to consult ophthalmology should instead be the focus for presentations that are less frequent. This is a potential area of opportunity for published ophthalmology education programs for emergency medicine residents to increase their applicability.[24] Furthermore, there is the opportunity to introduce measures that improve the exposure to ophthalmology at the medical school level. Potential conduits to facilitate the goal of basic clinical skills teaching and approaches to common ophthalmic complaints include didactic teaching, synchronous (interactive) learning, asynchronous learning and mandatory short term clinical rotations. [5, 25]

A strength of this investigation is its size; to our knowledge this is the largest Canadian investigation assessing ophthalmic presentations to the ED. This helps to address a gap in the currently literature by providing Canadian data that can be utilized to better inform Canadian training programs and resource allocation assessments. Additionally, the results have been classified and presented according to ophthalmic subspecialty and trauma related presentations to further guide emergency medicine ophthalmology training. The limitations of this investigation are primarily due to the use of retrospective data from a database. Consequently, there is a risk of limited or missed information, as well as the lack of pertinent outcomes such as visual acuity, the number of ER visits that required specialist referrals, and information pertaining to the patients’ prognosis. Lastly, a further limitation is that the ophthalmic diagnoses were based on the ED physician and were not confirmed by an eyecare professional. As a result of these limitations, future studies are required to determine the specific breakdown of ophthalmic presentations by subspecialty, and the urgency of the various presentations to the EDs. This present study can be used as hypothesis generating to inform these future investigations.

## Conclusion

As emergency medicine training programs shift to competency based medical education around the world, understanding ophthalmic related ED presentations is crucial to bridge the gap between basic knowledge and clinical practice. The present investigation summarizes ophthalmic presentation data over a 5-year period from all EDs in Ontario. Nearly 800,000 ophthalmic presentations have been summarized and classified by ophthalmic subspecialty and by trauma related presentations. Presentations pertaining to corneal and external diseases comprised over 50% of patient complaints. These results can help guide ophthalmic related knowledge translation. Future investigations can build on these results by assessing the impact of incorporating these findings into medical education programs and in improving resource allocation. Additionally, these results highlight that in Canadian EDs, a significant proportion of ophthalmic presentations are nonurgent; systems level efforts to improve access for eye-related complaints to healthcare professionals outside of the ED can help provide more cost-effective care and make ED resources more available for truly emergent ophthalmic and medical issues. As we emerge from the COVID-19 pandemic, optimising the structure of patient care access is crucial to help alleviate the pressure from overburdened EDs while effectively meeting patient healthcare needs.

## Electronic supplementary material

Below is the link to the electronic supplementary material.

Supplementary Material 1

Supplementary Material 2

Supplementary Material 3

Supplementary Material 4

## Data Availability

All data generated or analysed during this study are published in this article and the supplementary files.

## List of abbreviations

ED: Emergency department
EM: Emergency medicine
CIHI: Canadian Institute for Health Information
ICD-10: International Classification of Diseases 10th Revision
CTAS: Canadian Triage and Acuity Scale
SD: Standard Deviation
