# Supplementary material for: Profile of eye-related emergency department visits in Ontario – a Canadian perspective
Source: BMC Ophthalmol. 2023 Jul 10;23:305. doi: 10.1186/s12886-023-02999-x (PMC10332020; doi:10.1186/s12886-023-02999-x)
Supplement: Supplementary file 1 — Supplementary Material 1 [file 12886_2023_2999_MOESM1_ESM.pdf]

Supplemental Table 1. Frequency of Each Primary Ophthalmic Problem in Adult Cohort

ICD-10 Code	Description	Likely Emergent?	Frequency of Primary Problem
T159	Foreign body on external eye, unspecified part	Yes	100,152
H109	Conjunctivitis , unspecified	No	86,015
S050	Injury of conjunctiva and corneal abrasion without mention of foreign body	Yes	83480
H539	Visual disturbance, unspecified	Could not determine	50,392
H000	Hordeolum or Stye	No	29,715
H113	Conjunctival haemorrhage	No	24778
H571	Ocular pain	Could not determine	23759
H578	Other specified disorders of eye and adnexa	Could not determine	23286
S059	Injury of eye and orbit, unspecified	Could not determine	11608
H332	Serous retinal detachment	No	10789
H433	Other vitreous opacities	No	10231
H209	Iridocyclitis, unspecified	Could not determine	9696
H050	Acute inflammation of orbit (abscess, cellulitis, osteomyelitis, periostitis, tenonitis)	Yes	9399
S058	Other injuries of eye and orbit	Could not determine	9135
H169	Keratitis, unspecified	Could not determine	8846
H579	Disorder of eye and adnexa, unspecified	Could not determine	8534
H160	Corneal ulcer	Yes	8278
H010	Blepharitis	No	8032
H101	Acute atopic conjunctivitis	No	7952
H108	other conjunctivitis	No	7943
H438	Other disorders of virtreous body	Could not determine	7829
B309	Viral conjunctivitis (unspecified)	No	7231
H532	Diplopia	Could not determine	6134
H531	Subjective visual disturbances	No	5994
H103	Acute conjunctivitis unspecified	No	4849
H001	Chalazion	No	4663
S0110	Open wound eyelid & periocular area unco	Yes	4436
B023	Zoster ocular disease	Could not determine	4185
T151	Foreign body in conjunctival sac	Yes	3789
S051	Contusion of eyeball and orbital tissues	Yes	3449
H431	Vitreous haemorrhage	Yes	3210
T264	Burn of eye and adnexa part unspecified	Yes	3198
H409	Glaucoma unspecified	Could not determine	3098
H180	Corneal pigmentations and deposits	No	3078
T158	FB in oth & multiple parts external eye	Yes	2615
H151	Episcleritis	No	2474
H333	Retinal breaks without detachment	Yes	2442
T269	Corrosion of eye and adnexa part unspec	Yes	2339
B005	Herpesviral ocular disease	Could not determine	2199
H168	Other keratitis	Could not determine	2002
H100	Mucopurulent conjunctivitis	No	1906
H188	Other specified disorders of cornea	Could not determine	1813
H335	Other retinal detachments	Yes	1747
H162	Keratoconjunctivitis	No	1681
H534	Visual field defects	Could not determine	1653
H46	Optic neuritis	Could not determine	1630
H028	Other specified disorders of eyelid (included Hypertrichosis of eyelid or retained foregin body in eyelid)	Could not determine	1627
H043	Acute and unspecified inflammation of lacrimal passages	Yes	1385
S055	Penetrating wound of eyeball with foreign body	Yes	1269
H161	Other superficial keratitis without conjunctivitis	No	1254
H402	Primary angle-closure glaucoma	Yes	1225
H019	Inflammation of eyelid, unspecified	No	1223
H150	Scleritis	Yes	1181
H041	Other disorders of lacrimal gland	Could not determine	1088
S002	Other superficial injuries of eyelid and periocular area	Yes	1051
S053	Ocular laceration without prolapse or loss of intraocular tissue	Yes	1022
H269	Cataract, unspecified	No	978
S001	Contusion of eyelid and periocular area	Yes	930
H110	Pterygium	No	929
T266	Corrosion of cornea and conjunctival sac	Yes	927
H570	Anomalies of pupillary function	Could not determine	897
S02300	Fracture of orbital floor, closed	Yes	876
H330	retinal detachment with retinal break	Yes	868
T261	Burn of cornea and conjunctival sac	Yes	816
G438	other migraine (includes ophthalmoplegic migraine and retinal migraine)	No	788
H102	other acute conjunctivitis	No	743
H114	Other conjunctial vascular disorders and cysts	No	699
H400	Glaucoma suspect	No	677
H549	Unspecified visual impairment (binocular)	Could not determine	668
H029	Disorder of eyelid, unspecified	No	661
H356	Retinal haemorrhage	Could not determine	654
H353	Degeneration of macula and posterior pole	No	634
H045	Stenosis and insufficiency of lacrimal passages	No	603
H492	Sixth (abducent) nerve palsy	Yes	594
H024	Ptosis of eyelid	Could not determine	576
H189	Disorders of cornea, unspecified	Could not determine	558
H471	Papilloedema, unspecified	Could not determine	479
H210	Hyphaema	Yes	475
H105	Blepharoconjunctivitis	No	445
H490	Third (oculomotor) nerve palsy	Yes	438
H440	Purulent endophthalmitis	Yes	430
H264	After-cataract	No	414
H342	Other retinal artery occlusions	Yes	399
H111	Conjunctival degenerations and deposits	No	394
H208	other iridocyclitis	Could not determine	392
H348	Other retinal vascular occlusions	Could not determine	381
B300	Keratoconjunctivitis due to adenovirus	No	361
H359	Retinal disorder, unspecified	Could not determine	348
H5988	Other post procedural disorders of eye and adnexa	Could not determine	347
H011	Noninfectious dermatoses of eyelid	No	337
S054	Penetrating wound of orbit with or without foreign body	Yes	336
H118	other specified disorders of conjunctiva	No	336
H182	Other corneal oedema	No	331
H441	Other endophthalmitis	Yes	323
H341	Central retinal artery occlusion	Yes	308
Z010	Examination of eyes and vision	Could not determine	303
H119	disorders of conjunctiva, unspecified	No	289
H470	Disorders of optic nerve, not elsewhere classified	Could not determine	288
H158	Other disorders of sclera	No	283
B301	Conjunctivitis due to adenovirus	No	260
H018	Other specified inflammation of eyelid	No	243
H200	Acute and subacute iridocyclitis	Yes	238
H439	Disorder of vitreous body, unspecified	Could not determine	232
T260	Burn of eyelid and periocular area	Yes	212
T268	Corrosion of other parts of the eye and adnexa	Yes	212
H020	Entropion and trichiasis of eyelid	No	209
H599	Post procedural disorder of eye and adnexa, unspecified	Could not determine	208
E1433	Unspecified diabetes mellitus with other retinopathy	No	196
T852	Mechanical complication of intraocular lens	Yes	196
E1136	Type 2 diabetes mellitus with advanced ophthalmic disease	No	194
H040	Dacryoadentitis	Could not determine	190
H358	Other specified retinal disorders	No	189
H544	Blindness, monocular	Could not determine	188

H058	Other disorders of orbit (cyst of orbit)	No	178
S056	Penetrating wound of eyeball without foreign body	Yes	171
E1133	Type 2 diabetes mellitus with other retinopathy	No	167
E1430	Unspecified diabetes mellitus with background retinopathy	No	165
H350	Background retinopathy retinal vasc change	No	159
H533	Other disorders of binocular vision	Could not determine	154
H55	Nystagmus and other irregular eye movements	No	154
H401	Primary open-angle glaucoma	No	149
H546	Moderate visual impairment, monocular	Could not determine	147
H448	Other disorders of globe	Could not determine	147
H408	Other glaucoma	Could not determine	144
T263	Burn of other parts of eye and adnexa	Yes	142
B303	Acute epidemic haemorrhagic conjunctitivitis (enteroviral)	No	139
E1138	Type 2 diabetes with other specified complication not elsewhere classified (includes iritis, retinitis)	Could not determine	139
S0111	Open wound of eyelid and periocular area, complicated	Yes	135
H051	Chronic inflammatory disorders of orbit (granuloma, orbital inflammatory syndrome)	No	133
S052	Ocular laceration and rupture with prolapse or loss of intraocular tissue	Yes	132
E1130	Type 2 diabetes mellitus with background retinopathy	No	126
D231	Other benign neoplasm of skin of eyelid, including canthus	Could not determine	124
H545	Severe visual impairment, monocular	Could not determine	121
T200	Burn of unspecified degree of head and neck (includes eye with other parts of face)	Yes	121
H446	Retained (old) intraocular foreing body, magnetic	No	120
H540	Blindness, binocular	Could not determine	118
H159	Disorder of sclera, unspecified	Could not determine	116
H163	Interstitial and deep keratitis	No	114
H357	Separation of retinal layers	Yes	111
C441	Malignant neoplasm skin of eyelid, including canthus	Could not determine	106
H271	dislocation of lens	Yes	105
E1140	Type 2 diabetes with mononeuropathy (includes ophthalmoplegia, and others)	No	103
H185	Hereditary corneal dystrophies	No	103
E1436	Unspecified diabetes mellitus with advanced ophthalmic disease	No	102
H179	Corneal scar and opacity, unspecified	Could not determine	102
H059	Disorder of orbit, unspecified	Could not determine	98
H334	Traction detachment of retina	No	95
H521	Myopia	No	95
H104	Chronic conjunctivitis	No	93
H048	Other disorders of lacrimal system	Could not determine	91
M350	Sicca syndrome (includes syndrome with keratoconjunctivitis and others)	No	89
H499	Paralytic strabismus, unspecified	No	86
H527	Disorder of refraction, unspecified	No	80
H309	Chorioretinal inflammation, unspecified	Could not determine	79
H491	Fourth [trochlear] nerve palsy	Yes	78
T201	Burn of first degree of head and neck	Yes	78
H021	Ectropion of eyelid	No	77
E1432	Unspecified diabetes mellitus with proliferative retinopathy	No	76
T265	Corrosion of eyelid and periocular area	Yes	70
T853	Mechanical complication of other ocular prosthetic devices, implants and grafts (includes corneal grafts and prosthetic orbit of eye	Could not determine	68
H449	Disorder of globe, unspecified	Could not determine	65
H512	Internuclear ophthalmoplegia	Yes	65
B308	Other viral conjunctivitis (H13.1*)	No	63
H509	Strabismus, unspecified	Could not determine	62
T202	Burn of second degree of head and neck	No	62
E1033	Type 1 diabetes mellitus with other retinopathy	No	61
H354	Peripheral retinal degeneration	No	61
H042	Epiphora	No	59
H049	Disorder of lacrimal system, unspecified	Could not determine	57
H542	Moderate visual impairment, binocular	Could not determine	57
H023	Blepharochalasis	No	55
E1030	Type 1 diabetes mellitus with background retinopathy	No	51
H447	Retained (old) intraocular foreign body, nonmagnetic	No	51
H590	Keratopathy (bullous aphakic) following cataract surgery	No	51
H518	Other specified disorders of binocular movement	Could not determine	50
H178	Other corneal scars and opacities	Could not determine	49
H331	Choroidal degeneration	No	46
E1038	Type 1 diabetes mellitus with other specified ophthalmic complication not elsewhere classified	No	43
H187	Other corneal deformities	No	43
H349	Retinal vascular occlusion unspecified	Could not determine	43
L82	Seborrhoeic keratosis (could be on eyelid)	No	43
H5980	Cataract (lens) fragments in eye following cataract surgery	No	42
A740	Chlamydial conjunctivitis	No	41
H186	Keratoconus	No	41
E1440	Unspecified diabetes mellitus with mononeuropathy (includes Ophthalmoplegia)	No	39
D221	Malenocytic naevi of eyelid, including canthus	No	38
H432	Crystalline deposits in vitreous body	No	38
H218	Other specified disorders of iris and ciliary body	No	37
T204	Corrosion of unspecified degree of head and neck	Could not determine	36
H215	Other adhesions and disruptions of iris and ciliary body	No	35
H530	Amblyopia ex anopsia	No	35
H405	Glaucoma secondary to other eye disorders	No	34
H525	Disorders of accommodation	No	34
H026	Xanthelasma of eyelid	No	33
H025	Other disorders affecting eyelid function	Could not determine	32
H268	other specified cataract	No	31
A543	Gonococcal infection of eye	No	30
H443	Other degenerative disorders of globe	No	28
H430	Vitreous prolapse	Yes	27
H211	Other vascular disorders of iris and ciliary body	Could not determine	26
H403	Glaucoma secondary to eye trauma	Yes	26
H524	Presbyopia	No	26
S057	Avulsion of eye	Yes	26
D210	Other benign neoplasm of connective and other soft tissue of head, face and neck (includes eyelid)	No	24
E1036	Type 1 diabetes mellitus with advanced ophthalmic disease	No	24
H044	Chronic inflammation lacrimal passages	No	24
H184	Corneal degeneration	No	24
H318	Other specified disorders of choroid	No	24
C699	Malignant neoplasm of eye unspecified	Could not determine	23
H181	Bullous keratopathy	No	23
H259	Senile cataract unspecified	No	22
H510	Palsy of conjugate gaze	Yes	22
B580	Toxoplasma oculopathy	Could not determine	21
H473	Other disorders of optic disc	Could not determine	21
H477	Disorder of visual pathways unspecified	Could not determine	21
E1132	Type 2 diabetes mellitus with proliferative retinopathy	No	20
G360	Neuromyelitis optica	Could not determine	20
H183	Changes in corneal membranes	No	19
H313	Choroidal haemorrhage and rupture	Yes	19
L980	Pyogenic granuloma (could be related to eye area)	No	19
H500	Convergent concomitant strabismus	Could not determine	18
H498	Lens fragments in eye foll cataract surg	Could not determine	17
H520	Hypermetropia	No	16
S0408	Other and unspecified injury of optic nerve and pathways	Could not determine	16
H055	Retained (old) foreign body following penetrating wound of orbit	No	15
H404	Glaucoma secondary to eye inflammation	Yes	15
H501	Divergent concomitant strabismus	Could not determine	15

D480	Neoplasm of uncertain or unknown behaviour of bone or articular cartilage (includes connective tissue of eyelid, and other face parts)	Could not determine	14
H027	Other degenerative disorders of eyelid and periocular area	No	14
H164	Corneal neovascularization	Could not determine	14
H278	Other specified disorders of lens	Could not determine	14
H355	Hereditary retinal dystrophy	No	14
H505	Heterophoria	No	14
H519	Disorder of binocular movement, unspecified	Could not determine	14
B302	Viral pharyngoconjunctivitis	No	13
H213	Cyst iris ciliary body anterior chamber	Could not determine	13
H541	Moderate visual impairment binocular	Could not determine	13
H202	Lens-induced iridocyclitis	No	12
H310	Chorioretinal scars	No	12
H475	Disorders of other visual pathways	Could not determine	12
H535	Colour vision deficiencies	No	12
S02301	Fx orbital floor, open	Yes	12
H046	Other changes in lacrimal passages	No	11
H261	Traumatic cataract	Yes	11
H522	Astigmatism	No	11
H279	disorder of lens, unspecified	Could not determine	10
H508	Other specified strabismus	Could not determine	10
H526	Other disorders of refraction	No	10
C690	Malignant neoplasm of conjunctiva	No	9
D310	Benign neoplasm of conjunctiva	No	9
D319	Benign neoplasm of eye, unspecified	No	9
H201	Chronic iridocyclitis	No	9
H444	Hypotony of eye	Could not determine	9
H112	Conjunctival scars	No	8
H262	Complicated cataract	No	8
H340	Transient retinal artery occlusion	Yes	8
H5981	Cystoid Macular oedema foll cataract srg	No	8
C696	Malignant neoplasm of orbit	Could not determine	7
E1035	Type 1 diabetes mellitus with diabetic cataract	No	7
H314	Choroidal detachment	Yes	7
H352	Other proliferative retinopathy	No	7
C693	Malignant neoplasm of choroid	Could not determine	6
C694	Malignant neoplasm of ciliary body	Could not determine	6
H406	Glaucoma secondary to drugs	Could not determine	6
H511	Convergence insufficiency and excess	No	6
Q103	Other congenital malformations of eyelid	No	6
S0400	Laceration of optic nerve and pathways	Yes	6
C431	Malignant melanoma of eyelid, including canthus	Could not determine	5
D316	Benign neoplasm of orbit unspecified	No	5
E1032	Type 1 diabetes mellitus with proliferative retinopathy	No	5
H270	Aphakia	No	5
H308	Other chorioretinal inflammations	Could not determine	5
H442	Degenerative myopia	No	5
T205	Corrosion of first degree of head and neck	Yes	5
H022	Lagophthalmos	No	4
H476	Disorders of visual cortex	Yes	4
H506	Mechanical strabismus	No	4
Q121	Congenital displaced lens	No	4
Q141	Congenital malformation of retina	No	4
Q159	Congenital malformation of eye, unspecified	No	4
C490	Malignant neoplasm of connective and soft tissue of head, face and neck (includes eyelid, excludes orbit	Could not determine	3
C695	Malignant neoplasm lacrimal gland & duct	Could not determine	3
D311	Benign neoplasm of cornea	No	3
D313	Benign neoplasm of choroid	No	3
H053	Deformity of orbit	Could not determine	3
H171	Other central corneal opacity	Could not determine	3
H212	Degeneration of iris and ciliary body	No	3
H214	Pupillary membranes	No	3
H302	Posterior cyclitis	No	3
H445	Degenerated conditions of globe	Could not determine	3
H502	Vertical strabismus	No	3
H503	Intermittent heterotropia	No	3
M352	Behçet's disease (symptoms can include eye inflammation)	Could not determine	3
Q120	Congenital cataract	No	3
T262	Burn with resulting rupture and destruction of eyeball	Yes	3
C4100	Malignant neoplasm of craniofacial bones (includes Orbital)	Could not determine	2
C692	Malignant neoplasm of retina	Could not determine	2
E1336	Other specified diabetes mellitus with advanced ophthalmic disease	No	2
H258	Other senile cataract	No	2
H311	Vertical strabismus	No	2
H351	Choroidal degeneration	No	2
H543	Mild no visual impairmt binocular	No	2
Q100	Congenital ptosis	No	2
Q132	Other congenital malformations of iris	No	2
Q138	Oth congen malform ant segment eye	No	2
Q158	Oth spec congenital malformations of eye	No	2
C691	Malignant neoplasm of cornea	Could not determine	1
D312	Benign neoplasm of retina	Could not determine	1
D314	Benign neoplasm of ciliary body	Could not determine	1
D315	Benign neoplasm lacrimal gland & duct	Could not determine	1
H319	Benign neoplasm of eye unspecified	Could not determine	1
H474	Disorders of optic chiasm	Could not determine	1
H504	Other and unspecified heterotropia	No	1
Q106	Oth congen malformations lacrimal app	No	1
Q110	Cystic eyeball	No	1
Q111	Other anophthalmos	No	1
Q139	Congen malform ant segment eye NOS	No	1
Q140	Congenital malformation vitreous humour	No	1
Q143	Congenital malformation of choroid	No	1
Q150	Congenital glaucoma	Yes	1
T267	Corrosion w rupture/destruction eyeball	Yes	1
