# Supplementary material for: Profile of eye-related emergency department visits in Ontario – a Canadian perspective
Source: BMC Ophthalmol. 2023 Jul 10;23:305. doi: 10.1186/s12886-023-02999-x (PMC10332020; doi:10.1186/s12886-023-02999-x)
Supplement: Supplementary file 2 — Supplementary Material 2 [file 12886_2023_2999_MOESM2_ESM.pdf]

Supplemental Table 2. Frequency of Each Primary Ophthalmic Problem in Pediatric Cohort

ICD-10 Code	Description	Likely Emergent?	Percentage of Primary Problem
H109	Conjunctivitis, unspecified	No	29.16%
S050	Injury of conjunctiva and corneal abrasion without mention of foreign body	Yes	9.18%
H050	Acute inflammation of orbit (abscess, cellulitis, osteomyletis, periostitis, tenonitis)	Yes	4.85%
H000	Hordeolum and other deep inflammation of eyelid (includes abscess, furuncle and stye of eyelid)	No	4.81%
H578	Other specified disorders of eye and adnexa	Could not determine	4.42%
B309	Viral conjunctivitis (unspecified)	No	3.94%
S059	Injury of eye and orbit, unspecified	Could not determine	3.78%
T159	Foreign body on external eye, unspecified part	Yes	3.46%
H108	other conjunctivitis	No	3.18%
H101	Acute atopic conjunctivitis	No	2.74%
S0110	Open wound of eyelid and periocular area, uncomplicated	Yes	2.58%
H571	Ocular pain	Could not determine	2.35%
H103	Acute conjunctivitis, unspecified	No	2.30%
S058	Other injuries of eye and orbit	Could not determine	2.15%
H579	Disorder of eye and adnexa, unspecified	Could not determine	1.52%
H010	Blepharitis	No	1.44%
T150	Foreign body in cornea	Yes	1.39%
H113	Conjunctival haemorrhage	No	1.27%
H100	Mucopurulent conjunctivitis	No	1.26%
H045	Stenosis and insufficiency of lacrimal passages	No	1.21%
S002	Other superficial injuries of eyelid and periocular area	Yes	1.06%
H001	Chalazion	No	0.9-0.99%
S051	Contusion of eyeball and orbital tissues	Yes	0.8-0.89%
H539	Visual disturbance, unspecified	Could not determine	0.8-0.89%
H538	Other visual disturbances	Could not determine	0.5-0.59%
H028	Other specified disorders of eyelid (included Hypertrichosis of eyelid or retained foreign body in eyelid)	Could not determine	0.3-0.39%
S001	Contusion of eyelid and periocular area	Yes	0.3-0.39%
T151	Foreign body in conjunctival sac	Yes	0.3-0.39%
H019	Inflammation of eyelid, unspecified	No	0.3-0.39%
H043	Acute and unspecified inflammation of lacrimal passages	Yes	0.3-0.39%
H532	Diplopia	Could not determine	0.3-0.39%
H169	Keratitis, unspecified	Could not determine	0.3-0.39%
H102	other acute conjunctivitis	No	0.2-0.29%
T269	Corrosion of eye and adnexa, prt unspecified	Yes	0.2-0.29%
H209	Irodocyclitis, unspecified	Could not determine	0.2-0.29%
H160	Corneal ulcer	Yes	0.2-0.29%
H531	Subjective visual disturbances	Could not determine	0.2-0.29%
S053	Ocular laceration without prolapse or loss of intraocular tissue	Yes	0.1-0.19%
P391	Neonatal conjunctivitis and dacryocystitis	Could not determine	0.1-0.19%
H151	Episcleritis	Could not determine	0.1-0.19%
B005	Herpesviral ocular disease	Could not determine	0.1-0.19%
T158	Foreign body in other and multiple parts of external eye	Yes	0.1-0.19%
H052	Exophthalmic conditions	Could not determine	0.1-0.19%
H509	Strabismus, unspecified	Could not determine	0.1-0.19%
H570	Anomalies of pupillary function	Could not determine	0.1-0.19%
T264	Burn of eye and adnexa, part unspecified	Yes	0.1-0.19%
H029	Disorder of eyelid, unspecified	Could not determine	0.1-0.19%
Q105	Congenital stenosis and stricture of lacrimal duct	No	0.1-0.19%
H471	Papilloedema, unspecified	Could not determine	0.1-0.19%
B023	Zoster ocular disease	Could not determine	0.1-0.19%
H105	Blepharoconjunctivitis	No	0.1-0.19%
T266	Corrosion of cornea and conjunctival sac	Yes	0.01-0.09%
H500	Convergent concomitant strabismus	Could not determine	0.01-0.09%
H114	Other conjunctival vascular disorders and cysts	No	0.01-0.09%
S055	Penetrating wound of eyeball with foreign body	Yes	0.01-0.09%
H210	Hyphaema	Yes	0.01-0.09%
H162	Keratoconjunctivitis	Could not determine	0.01-0.09%
S02300	Fracture of orbital floor, closed	No	0.01-0.09%
H018	Other specified inflammation of eyelid	Could not determine	0.01-0.09%
H46	Optic neuritis	Could not determine	0.01-0.09%
H168	Other keratitis	Could not determine	0.01-0.09%
H011	Noninfectious dermatoses of eyelid	No	0.01-0.09%
H118	other specified disorders of conjunctiva	No	0.01-0.09%
H119	disorders of conjunctiva, unspecified	No	0.01-0.09%
H058	Other disorders of orbit (cyst of orbit)	Could not determine	0.01-0.09%
H433	Other vitreous opacities	No	0.01-0.09%
T261	Burn of cornea and conjunctival sac	Yes	0.01-0.09%
H188	Other specified disorders of cornea	Could not determine	0.01-0.09%
H55	Nystagmus and other irregular eye movements	Could not determine	0.01-0.09%
H041	Other disorders of lacrimal gland	Could not determine	0.01-0.09%
S054	Penetrating wound of orbit with or without foreign body	Yes	0.01-0.09%
G438	other migraine (includes ophthalmoplegic migraine and retinal migraine)	Could not determine	0.01-0.09%
H332	Serous retinal detachment	Yes	0.01-0.09%
H048	Other disorders of lacrimal system	Could not determine	0.01-0.09%
H110	Pterygium	No	0.01-0.09%
H180	Corneal pigmentations and deposits	No	0.01-0.09%
S0111	Open wound of eyelid and periocular area, complicated	Yes	0.01-0.09%
H024	Ptosis of eyelid	No	0.01-0.09%
B301	Conjunctivitis due to adenovirus	No	0.01-0.09%
H534	Visual field defects	Could not determine	0.01-0.09%
T260	Burn of eyelid and periocular area	Yes	0.01-0.09%
H158	Other disorders of sclera	Could not determine	0.01-0.09%
H161	Other superficial keratitis without conjunctivitis	No	0.01-0.09%
H189	Disorders of cornea, unspecified	Could not determine	0.01-0.09%
H049	Disorder of lacrimal system, unspecified	Could not determine	0.01-0.09%
H549	Unspecified visual impairment (binocular)	Could not determine	0.01-0.09%
B308	Other viral conjunctivitis (H13.1*)	No	0.01-0.09%
H441	Other endophthalmitis	Yes	0.01-0.09%
H059	Disorder of orbit, unspecified	Could not determine	0.01-0.09%
H150	Scleritis	Yes	0.01-0.09%
H492	Sixth (abducent) nerve palsy	Yes	0.01-0.09%
H040	Dacryoadentitis	Could not determine	0.01-0.09%
H111	Conjunctival degenerations and deposits	No	0.01-0.09%
S056	Penetrating wound of eyeball without foreign body	Yes	0.01-0.09%
H521	Myopia	No	0.01-0.09%
Z010	Examination of eyes and vision	No	0.01-0.09%
H208	other iridocyclitis	Could not determine	0.01-0.09%
H518	Other specified disorders of binocular movement	Could not determine	0.01-0.09%
H440	Purulent endophthalmitis	Yes	0.01-0.09%
H051	Chronic inflammatory disorders of orbit (granuloma, orbital inflammatory syndrome)	No	0.01-0.09%
H409	Glaucoma, unspecified	Could not determine	0.01-0.09%
H503	Intermittent heterotropia	No	0.01-0.09%
H473	Other disorders of optic disc	Could not determine	0.01-0.09%
H530	Amblyopia ex anopsia	No	0.01-0.09%

H431	Vitreous haemorrhage	Yes	0.01-0.09%
H333	Retinal breaks without detachment	Yes	0.01-0.09%
S052	Ocular laceration and rupture with prolapse or loss of intraocular tissue	Yes	0.01-0.09%
H104	Chronic conjunctivitis	No	0.01-0.09%
H159	Disorder of sclera, unspecified	Could not determine	0.01-0.09%
T263	Burn of other parts of eye and adnexa	Yes	0.01-0.09%
H020	Entropion and trichiasis of eyelid	No	0.01-0.09%
H527	Disorder of refraction, unspecified	No	0.01-0.09%
L980	Pyogenic granuloma (could be related to eye area)	No	0.01-0.09%
H269	Cataract, unspecified	No	0.01-0.09%
T268	Corrosion of other parts of the eye and adnexa	Yes	0.01-0.09%
T853	Mechanical complication of other ocular prosthetic devices, implants and grafts (includes corneal grafts and prosthetic orbit of eye	Could not determine	0.01-0.09%
H335	Other retinal detachments	Yes	0.01-0.09%
H438	Other disorders of vitreous body	Could not determine	0.01-0.09%
C692	Malignant neoplasm of retina	Could not determine	0.01-0.09%
H163	Interstitial and deep keratitis	No	0.01-0.09%
H505	Heterophoria	No	0.01-0.09%
T202	Burn of second degree of head and neck	Yes	0.01-0.09%
B300	Keratoconjunctivitis due to adenovirus	No	0.01-0.09%
H5988	Other postprocedural disorders of eye and adnexa	Could not determine	0.01-0.09%
T200	Burn of unspecified degree of head and neck (includes eye with other parts of face)	Yes	0.01-0.09%
B303	Acute epidemic haemorrhagic conjunctivitis (enteroviral)	No	0.01-0.09%
H182	Other corneal oedema	No	0.01-0.09%
H309	Chorioretinal inflammation, unspecified	Could not determine	0.01-0.09%
H470	Disorders of optic nerve, not elsewhere classified	Could not determine	0.01-0.09%
T201	Burn of first degree of head and neck	Yes	0.01-0.09%
T265	Corrosion of eyelid and periocular area	Yes	0.01-0.09%
B302	Viral pharyngoconjunctivitis	No	0.01-0.09%
H356	Retinal haemorrhage	Could not determine	0.01-0.09%
H027	Other degenerative disorders of eyelid and periocular area	No	0.01-0.09%
H042	Epiphora	No	0.01-0.09%
H046	Other changes in lacrimal passages	Could not determine	0.01-0.09%
H330	retinal detachment with retinal break	Yes	0.01-0.09%
H501	Divergent concomitant strabismus	No	0.01-0.09%
H525	Disorders of accommodation	No	0.01-0.09%
Q120	Congenital cataract	No	0.01-0.09%
H218	Other specified disorders of iris and ciliary body	No	0.01-0.09%
H350	Background retinopathy and retinal vascular changes	No	0.01-0.09%
H400	Glaucoma suspect	No	0.01-0.09%
H449	Disorder of globe, unspecified	Could not determine	0.01-0.09%
H499	Paralytic strabismus, unspecified	No	0.01-0.09%
H533	Other disorders of binocular vision	Could not determine	0.01-0.09%
A543	Gonococcal infection of eye	No	0.01-0.09%
H179	Corneal scar and opacity, unspecified	No	0.01-0.09%
H358	Other specified retinal disorders	No	0.01-0.09%
H448	Other disorders of globe	Could not determine	0.01-0.09%
H535	Colour vision deficiencies	No	0.01-0.09%
H599	Postprocedural disorder of eye and adnexa, unspecified	Could not determine	0.01-0.09%
Q150	Congenital glaucoma	Could not determine	0.01-0.09%
T204	Corrosion of unspecified degree of head and neck	Yes	0.01-0.09%
H200	Acute and subacute iridocyclitis	Yes	<0.01%
H508	Other specified strabismus	No	<0.01%
H519	Disorder of binocular movement, unspecified	Could not determine	<0.01%
H544	Blindness, monocular	Could not determine	<0.01%
A740	Chlamydial conjunctivitis	No	<0.01%
D231	Other benign neoplasm of skin of eyelid, including canthus	No	<0.01%
H187	Other corneal deformities	Could not determine	<0.01%
H512	Internuclear ophthalmoplegia	Yes	<0.01%
H522	Astigmatism	No	<0.01%
H546	Moderate visual impairment, monocular	Could not determine	<0.01%
Q103	Other congenital malformations of eyelid	Could not determine	<0.01%
D221	Malenocytic naevi of eyelid, including canthus	No	<0.01%
H025	Other disorders affecting eyelid function	No	<0.01%
H044	Chronic inflammation of lacrimal passages	No	<0.01%
H359	Retinal disorder, unspecified	No	<0.01%
H403	Glaucoma secondary to eye trauma	Yes	<0.01%
H540	Blindness, binocular	Could not determine	<0.01%
H590	Keratopathy (bullous aphakic) following cataract surgery	No	<0.01%
Q106	Other congenital malformations of lacrimal apparatus	No	<0.01%
Q130	Coloboma of iris	No	<0.01%
Q131	Absence of iris	No	<0.01%
Q132	Other congenital malformations of iris	No	<0.01%
Q159	Congenital malformation of eye, unspecified	No	<0.01%
D319	Benign neoplasm of eye, unspecified	No	<0.01%
H023	Blepharochalasis	No	<0.01%
H185	Hereditary corneal dystrophies	No	<0.01%
H215	Other adhesions and disruptions of iris and ciliary body	No	<0.01%
H260	Infantile, juvenile and presenile cataract	No	<0.01%
H261	Traumatic cataract	Yes	<0.01%
H279	disorder of lens, unspecified	No	<0.01%
H353	Degeneration of macula and posterior pole	No	<0.01%
H490	Third (oculomotor) nerve palsy	Yes	<0.01%
H491	Fourth [trochlear] nerve palsy	Yes	<0.01%
H510	Palsy of conjugate gaze	Yes	<0.01%
H511	Convergence insufficiency and excess	No	<0.01%
H520	Hypermetropia	No	<0.01%
Q100	Congenital ptosis	No	<0.01%
Q104	Absence and agenesis of lacrimal apparatus	No	<0.01%
T205	Corrosion of first degree of head and neck	Yes	<0.01%
D310	Benign neoplasm of conjunctiva	No	<0.01%
D480	Neoplasm of uncertain or unknown behaviour of bone or articular cartilage (includes connective tissue of eyelid, and other face parts)	No	<0.01%
E1038	Type 1 diabetes mellitus with other specified ophthalmic complication not elsewhere classified	No	<0.01%
H021	Ectropion of eyelid	No	<0.01%
H053	Deformity of orbit (atrophy, exostosis)	No	<0.01%
H112	Conjunctival scars (symblepharon)	No	<0.01%
H264	After-cataract	No	<0.01%
H310	Chorioretinal scars (Includes: Macula scars of posterior pole (post-inflammatory)(post-traumatic), solar retinopathy)	No	<0.01%
H318	Other specified disorders of choroid	No	<0.01%
H319	Disorder of choroid, unspecified	No	<0.01%
H348	Other retinal vascular occlusions	Could not determine	<0.01%
H351	Retinopathy of prematurity	No	<0.01%
H355	Hereditary retinal dystrophy	No	<0.01%
H401	Primary open-angle glaucoma	No	<0.01%
H408	Other glaucoma	Could not determine	<0.01%
H432	Crystalline deposits in vitreous body	No	<0.01%
H439	Disorder of vitreous body, unspecified	Could not determine	<0.01%
H443	Other degenerative disorders of globe	No	<0.01%
H444	Hypotony of eye	Could not determine	<0.01%
H475	Disorders of other visual pathways	Could not determine	<0.01%
H477	Disorders of visual pathways, unspecified	Could not determine	<0.01%
H502	Vertical strabismus	No	<0.01%
