# Supplementary material for: Profile of eye-related emergency department visits in Ontario – a Canadian perspective
Source: BMC Ophthalmol. 2023 Jul 10;23:305. doi: 10.1186/s12886-023-02999-x (PMC10332020; doi:10.1186/s12886-023-02999-x)
Supplement: Supplementary file 3 — Supplementary Material 3 [file 12886_2023_2999_MOESM3_ESM.pdf]

Supplemental Table 3. Full Summary of all Trauma Related Presentations in Adult Cohort

ICD-10 Code	Code Description	Frequency of Ophthalmic Problem	Percentage of Ophthalmic Problem
T159	Foreign body on external eye, or Cornea	100,152	44.50%
S050	Corneal abrasion/Injury of conjunctiva	83,480	37.10%
S059	Injury of eye and orbit, unspecified (includes injury of eye NOS)	20,743	9.20%
T264	Ocular/Orbital burns	7,920	3.52%
S0110	Eyelid Abrasion and Laceration	5,487	2.40%
S051	Contusion/Blunt Trauma Injuries of the Eye and Orbit	4,479	2.00%
S0110	Open wound of eyelid, uncompl.	4,436	1.97%
S051	Contusion of eyeball and orbital tissues (includes corneal contusion, traumatic hyphaema)	3,449	1.53%
T264	Burn of eye and adnexa, part unspecified (includes welder's flash)	3,198	1.42%
S055	Penetrating wound of eyeball with foreign body	1,269	0.56%
S053	Ocular laceration without prolapse or loss of intraocular tissue	1022	0.45%
S02300	Fx orbital floor, closed	876	0.39%
S054	Penetrating wound of orbit with or without foreign body	337	0.15%
T268	Corrosion of other parts of eye and adnexa	212	0.08%
S056	Penetrating wound of eyeball without foreign body (includes ocular penetration NOS)	171	0.06%
S0111	Open wound of eyelid +FB/Infection	135	0.06%
S052	Ocular laceration and rupture with prolapse or loss of intraocular tissue	132	0.03%
H403	Glaucoma, secondary to eye trauma	26	0.01%
S057	Avulsion of eye (includes traumatic enucleation)	26	0.01%
H261	Traumatic cataract	11	<0.01%
S0400	Laceration of optic nerve and pathways	6	<0.01%
S041	Injury of oculomotor nerve	0	<0.01%
S042	Injury of trochlear nerve	0	<0.01%
S040	Injury of optic nerve and pathways (includes 2nd cranial nerve, optic chiasm, visual cortex)	0	<0.01%
T904	Sequelae of injury of eye and orbit	0	<0.01%
