# Supplementary material for: Profile of eye-related emergency department visits in Ontario – a Canadian perspective
Source: BMC Ophthalmol. 2023 Jul 10;23:305. doi: 10.1186/s12886-023-02999-x (PMC10332020; doi:10.1186/s12886-023-02999-x)
Supplement: Supplementary file 4 — Supplementary Material 4 [file 12886_2023_2999_MOESM4_ESM.pdf]

Supplemental Table 4. Full Summary of all Trauma Related Presentations in Pediatric Cohort

ICD-10 Code	Code Description	Frequency of Ophthalmic Problem	Percentage of Ophthalmic Problem
S050	Corneal abrasion/Injury of conjunctiva	11,074	7.40%
S059	Injury of eye and orbit, unspecified (includes injury of eye NOS)	4,555	3.04%
T159	Foreign body on external eye, part unspecified	4,170	2.79%
S0110	Open wound of eyelid, uncompl.	3,112	2.08%
S058	Other injuries of eye and orbit (includes lacrimal duct injury)	2,588	1.73%
T150	Foreign body in cornea	1,676	1.12%
S002	Other superficial injuries of eyelid and periocular area	1,284	0.86%
S051	Contusion of eyeball and orbital tissues (includes corneal contusion, traumatic hyphaema)	984	0.66%
S001	Contusion of eyelid and periocular area	463	0.31%
T151	Foreign body in conjunctival sac	430	0.29%
T269	Corrosion of eye and adnexa, part unspecified	283	0.19%
S053	Ocular laceration without prolapse or loss of intraocular tissue	217	0.14%
T158	Foreign body in other and multiple parts of external eye (includes foreign body in lacrimal punctum)	187	0.12%
T264	Burn of eye and adnexa, part unspecified (includes welder's flash)	164	0.11%
T266	Corrosion of cornea and conjunctival sac	112	0.07%
S055	Penetrating wound of eyeball with foreign body	102	0.07%
S02300	Fx orbital floor, closed	96	0.06%
T261	Burn of cornea and conjunctival sac	76	0.05%
S054	Penetrating wound of orbit with or without foreign body	69	0.05%
S0111	Open wound of eyelid +FB/Infection	56	0.04%
T260	Burn of eyelid and periocular area	49	0.03%
S056	Penetrating wound of eyeball without foreign body (includes ocular penetration NOS)	37	0.02%
S052	Ocular laceration and rupture with prolapse or loss of intraocular tissue	20	0.01%
T263	Burn of other parts of eye and adnexa	19	0.01%
T268	Corrosion of other parts of eye and adnexa	17	0.01%
T265	Corrosion of eyelid and periocular area	11	0.01%
H403	Glaucoma, secondary to eye trauma	4	0.00%
H261	Traumatic cataract	3	0.00%
S057	Avulsion of eye (includes traumatic enucleation)	2	0.00%
T262	Burn with resulting rupture and destruction of eyeball	1	0.00%
